# Linkage disequilibrium and past effective population size in native Tunisian cattle

**DOI:** 10.1590/1678-4685-GMB-2017-0342

**Published:** 2019-02-18

**Authors:** Slim Ben Jemaa, Nejia Thamri, Sofiane Mnara, Emmanuelle Rebours, Dominique Rocha, Mekki Boussaha

**Affiliations:** 1 National Institute of Agronomic Research of Tunisia National Institute of Agronomic Research of Tunisia Laboratoire des Productions Animales et Fourragères Ariana Tunisia National Institute of Agronomic Research of Tunisia, Laboratoire des Productions Animales et Fourragères, Ariana, Tunisia; 2 Livestock and Pasture Office Livestock and Pasture Office Tunis Belvedere Tunisia Livestock and Pasture Office, Tunis Belvedere, Tunisia; 3 National Bank of Genes of Tunisia National Bank of Genes of Tunisia Tunis Tunisia National Bank of Genes of Tunisia, Tunis, Tunisia; 4 Université Paris Saclay Université Paris Saclay AgroParisTech GABI Jouy-en-Josas France GABI, INRA, AgroParisTech, Université Paris Saclay, Jouy-en-Josas, France

**Keywords:** Tunisian cattle, conservation, linkage disequilibrium, effective population size, single nucleotide polymorphism

## Abstract

To carry out effective genome-wide association studies, information about linkage disequilibrium (LD) is essential. Here, we used medium-density SNP chips to provide estimates of LD in native Tunisian cattle. The two measures of LD that were used, mean *r*^2^ and *D’*, decreased from 0.26 to 0.05 and from 0.73 to 0.40, respectively, when the distance between markers increased from less than 20 Kb to 200 Kb. The decay in LD over physical distance occurred at a faster rate than that reported for European and other indigenous breeds, and reached background levels at less than 500 Kb distance. This is consistent with the absence of strong selective pressure within the Tunisian population and suggests that, in order to be effective, any potential genome-wide association mapping studies will need to use chips with higher marker density. An analysis of effective population size (Ne) based on LD data showed a decline in past Ne, with a sudden drop starting about eight generations ago. This finding, combined with the high levels of recent inbreeding revealed by runs of homozygosity (ROH) analysis, indicate that this population is endangered and may be in urgent need of a conservation plan that includes a well-designed genetic management program.

## Introduction

Native Tunisian cattle are small animals that are well adapted to the harsh environmental conditions that characterize their rearing area, such as reduced food resources, elevated temperatures during the hot season, and an abundance of parasites and pathogens. They are currently endangered by the result of extensive crossbreeding with imported breeds (Djemali *and* Kayouli, 2003). Local cattle in Tunisia belong to three main ecotypes, defined according to coat color. These are the Blonde du Cap Bon (BLCAP) (white), the Brune de l’Atlas Grise (BRATG) (grey), and the Brune de l’Atlas Fauve (BRATF) (tawny). Native Tunisian cattle have not been subjected to artificial selection, and are in need of breeding programs to improve their production performance. Nowadays, the availability of high-throughput genotyping technologies makes it possible to use genetic markers to map genes that are responsible for economically important traits. Such information can then be incorporated into breeding programs. Before one can efficiently map these genes, it is, however, necessary to first determine the marker density that is compatible with the distances across which linkage disequilibrium (LD) extends in the population of interest. LD refers to the non-random association of alleles at different loci in gametes. LD patterns vary substantially among populations and can arise both through physical linkage between loci and, more generally, through a variety of evolutionary forces that structure a genome, such as genetic drift, admixture, and natural selection. By characterizing the pattern of LD within a population, we are able to determine the amount of markers needed to optimize the mapping resolution of genetic variants related to economic traits. Furthermore, LD is used routinely for the study of the demographic history of populations in many species, such as humans (Reich *et al.*, 2001; McEvoy *et al.*, 2011), cattle (Khatkar *et al.*, 2008; Porto-Neto *et al.*, 2014), and sheep (McRae *et al.*, 2002; Al-Mamun *et al.*, 2015). It can also be used to infer recent and historical effective population size (Ne). Ne is defined as the size of an “ideal” population – a hypothetical population that is panmictic, large enough that sampling error is negligible (thereby preventing genetic drift), with an equal sex ratio, and without migration, mutation, or selection – that would experience the effects of inbreeding or drift to the same degree as observed in the actual breeding population (Falconer and Mackay, 1996). Ne is an important parameter for monitoring the genetic health of endangered populations as it reflects the risk of extinction that is posed by genetic drift (which drives the loss of alleles in endangered populations). Here, we characterized LD in the genome of local Tunisian cattle by analyzing 87 individuals using the Illumina BovineSNP50 chip assay. We then used LD estimates to infer changes in the past effective population size of native Tunisian cattle over time. In addition, to get an overview of population inbreeding, we present a genome-wide characterization of runs of homozygosity (ROH) in the Tunisian individuals.

## Materials and Methods

### Animal sampling

We identified for sampling 40 individuals of indigenous Tunisian cattle; these originated from both the northwestern and central parts of Tunisia, which are two of the main regions in the country where local cattle breeds are found. Animal sampling was carried out by a multi-institutional team representing the Livestock and Pasture Office (OEP), the Office of Silvopastoral Development of the North-West (ODESYPANO), the National Gene Bank (BNG), and the National Institute of Agronomic Research of Tunisia (INRAT).

Since most native cattle have been extensively crossed with imported breeds, significant efforts were made to select purebred individuals. Selection was performed based on morphological criteria and targeted isolated mountainous regions where no historical artificial insemination activity was recorded. Our sample included 25 BRATF and 15 BRATG individuals. Blood samples were collected in EDTA Vacutainer tubes under procedures approved by the Tunisian Veterinary Authority.

### DNA extraction and genotyping assays

DNA was extracted using the phenol-chloroform protocol (Cheng *et al.*, 1995). DNA quantity and quality were evaluated using a Nanodrop instrument, and DNA samples were then genotyped on the BovineSNP50 BeadChip Ver. 2 (Illumina, San Diego, CA, USA) at the Labogena core facility (Jouy-en-Josas, France) using standard operating procedures, as recommended by the manufacturer (http://www.illumina.com).

### Marker quality control and selection

Overall, we obtained genotyping data from all 40 animals for 52,274 SNPs. Quality control of these data was performed using PLINK software v1.9 (Purcell *et al.*, 2007). Samples with a genotyping rate of <90% across autosomal SNPs and markers with a call rate <90% were discarded. Using these criteria, we excluded one BRATF individual and 2,232 SNPs from further analysis. An exact test for Hardy-Weinberg equilibrium (*p* <0.01) was then carried out on the remaining SNPs using PLINK, which resulted in the retention of 49,362 SNPs for subsequent analyses. We combined these newly generated genotypes with those already available for 48 individuals of Tunisian cattle: 18 BRATF, 15 BRATG, and 15 BLCAP (Ben Jemaa *et al.*, 2015). Thus, our final data set was composed of 48,458 SNPs genotyped for 87 Tunisian individuals.

To evaluate the effect of minor allele frequencies (MAFs) on LD, we estimated the LD score using three different MAF thresholds: 0.01 (MAF 0.01), 0.05 (MAF 0.05), and 0.1 (MAF 0.1). The application of these three thresholds resulted in the retention of 42,699, 38,078, and 33,146 SNPs, respectively. In order to facilitate comparisons among the three datasets, we reduced the size of the MAF 0.01 and MAF 0.05 datasets to 33,333 and 33,509 SNPs, respectively.Marker coverage and SNP density per chromosome are indicated for each MAF threshold in Table S1, and the distribution of allele frequencies for each MAF cutoff value is illustrated in Figure S1.

### Detection of runs of homozygosity

To assess levels of inbreeding in our 87 individuals, we examined our genetic data for runs of homozygosity (ROH). These were detected in sliding windows of 20 SNPs using PLINK. No more than five missing calls and one heterozygous SNP were allowed in each window. The minimum length of a ROH segment was set to 1 Mb, and a ROH was declared if it contained at least 20 SNPs. The minimum required SNP density was one SNP per 200 kb and the maximum gap allowed between any two consecutive SNPs was 1000 kb. ROHs were classified into seven categories based on length (1 to 4.999 Mb, 5 to 9.999 Mb, 10 to 14.999 Mb, 15 to 19.999 Mb, 20 to 24.999 Mb, 25 to 49.999 Mb, >50 Mb).

### Estimation of linkage disequilibrium

We used the genotyping data from these 87 individuals to estimate linkage disequilibrium in our sample. For this, we used Haploview v4.2 (Barrett *et al.*, 2005) to generate two standard descriptive LD parameters, *D’* and *r*^*2*^*,* for all syntenic SNP pairs that were less than 50 Mb apart. All values of *r*^2^ between syntenic markers were corrected for sample size using the following equation:

$$r^{2}{}_{corrected}=\frac{r^{2}{}_{computed}-1/n}{1-1/n}$$

where n is the number of haplotypes in the sample (Villa-Angulo *et al.*, 2009). To better understand how LD estimates were related to physical distances between syntenic marker pairs, we used R software (https://www.r-project.org/) to analyze LD decay over the following windows of genetic distance (Kb): 0-19; 20-39; 40-59; 60-99; 100-199; 200-499; 500-999; 1,000-1,999; 2,000-4999; 5,000-9,999; 10,000-11,999; 12,000-14,999; 15,000-16,999; 17,000-19,999; 20,000-29,999; 30,000-50,000. Average *r*^2^ and *D’* values were determined and plotted for all combined autosomes for each distance window and for each MAF cutoff value. LD parameters and the percentage of SNP pairs with *r*^2^ values >0.2 or 0.8 were also calculated between adjacent SNPs within each chromosome. We further determined the amount of background linkage disequilibrium by calculating *r*^2^ and *D’* for non-syntenic SNPs. This was done by randomly selecting 1,700 SNPs (~5% of the markers) distributed over all chromosomes.

### Estimation of effective population size

We used SNeP software (Barbato *et al.*, 2015) to estimate Ne and its change over time using the observed extent of LD calculated for the three MAF thresholds. SNeP estimates the historic effective population size based on the following equation (Corbin *et al.*, 2012).

$$N_{T}\left(t\right)=\frac{1}{4f\left(c_{t}\right)}*\left[\frac{1}{E\left(r^{2}{}_{adj}\text{v}c_{t}\right)}-a\right]$$

where N _T_(t) is the effective population size estimated t generations ago, f(c_t_) is the mapping function used to estimate the recombination rate (c_t_) t generations ago, *r*^2^_adj_ is the LD estimation, adjusted for sampling bias (*r*^2^_adj_=*r*^2^-(1/2N), where N is the population sample size), and “a” is a constant that accounts for mutation. Inter-marker distances used in our N_T_(t) estimates ranged from 0.02 to 50 Mb. We also set a to 1 when f(c_t_) was calculated using the Sved and Feldman (1973) 0 approximation: f(c_t_) = d(1-d/2), where d is the linkage distance between SNPs, which is proportional to the physical distance, δ (δ=10^-8^*d).

## Results

### Detection of ROHs in the Tunisian cattle genome

A total of 3,313 ROHs were identified in the 87 Tunisian cattle individuals. The average number of ROHs per individual was 38 ± 12.6 (range: 16 to 81). Most ROHs (85%) were between 1 and 5 Mb long. Chromosome BTA01 had the highest number of ROHs (305), while BTA28 had the least (39) (Figure S2). Overall, the number of ROHs per chromosome tended to decrease with chromosome length (correlation between chromosome length and the number of ROHs was ~92%). Among the 87 individuals, 36 (~41%) had a total sum of ROHs >100 Mb, while 15 individuals (~ 17%) had a total sum of ROHs >250 Mb (Figure S3). Among these, 14 individuals (~93%) had at least one ROH that was more than 25 Mb long (Table S2). Approximately 5.4%, on average, of the entire genome of Tunisian cattle was covered by ROH segments. Chromosome BTA25 showed the highest coverage by ROHs (12.4%) while BTA01 had the least (1.6%) (Figure S2).

#### Characterization of Linkage Disequilibrium

**Figure 1:**
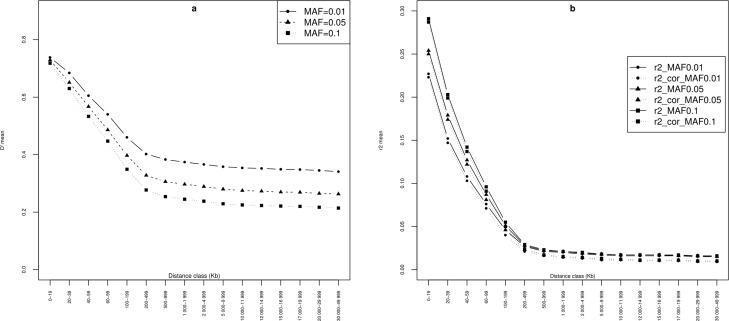
Average LD decay pooled over all autosomes for three different minor allele frequency (MAF) thresholds in the Tunisian cattle population: (a) *D’* , (b) *r*^*2*^ (as estimated by Haploview) and *r*^2^ corrected for sample size (*r*^2^_cor).

The extent of LD was first evaluated for all syntenic SNP pairs for all chromosomes combined. Table 1 shows the values estimated for the decay of *r*^2^, *r*^2^ corrected for sample size, and *D’* for all 29 autosomes; this information is displayed graphically in Figure 1. Correction for sample size resulted in a slight decrease (~7% on average) in *r*^2^ estimates for markers that were less than 500 Kb apart. Overall, LD parameters declined rapidly over short inter-marker distances. For example, the estimated *r*^2^ values averaged over the three MAF thresholds decreased from 0.26 for syntenic SNPs less than 20 Kb apart to 0.05 for those that were less than 200 Kb apart. However, LD decay was slower over longer physical distances. For example, mean *D’* dropped from 0.33 for SNPs separated by 200-499 Kb to 0.28 for those that were more than 30 Mb apart.

**Table 1:** Average *D’* and *r*^2^ estimates by distance class.

Distance_class (Kb)	Number of pairs			Average *D’* (sd)			Average *r*^2^ (sd)			Average *r*^2^ corrected (sd)		
	MAF0.01	MAF0.05	MAF0.1	MAF0.01	MAF0.05	MAF0.1	MAF0.01	MAF0.05	MAF0.1	MAF0.01	MAF0.05	MAF0.1
0-19	546	551	580	0.738(0.34)	0.729(0.33)	0.718(0.33)	0.227(0.3)	0.254(0.29)	0.291(0.31)	0.223(0.299)	0.250(0.29)	0.287(0.30)
20-39	11 888	12 264	12 074	0.684(0.35)	0.651(0.34)	0.630(0.34)	0.152(0.22)	0.179(0.24)	0.203(0.25)	0.147(0.226)	0.174(0.23)	0.199(0.25)
40-59	9 556	9 738	9 571	0.605(0.35)	0.567(0.34)	0.533(0.34)	0.108(0.18)	0.127(0.19))	0.142(0.2)	0.103(0.179)	0.122(0.19)	0.137(0.19)
60-99	19 319	19 759	19 398	0.540(0.36)	0.486(0.34)	0.447(0.32)	0.076(0.14)	0.087(0.14)	0.096(0.15)	0.071(0.137)	0.081(0.14)	0.091(0.15)
100-199	47 353	48 233	47 311	0.460(0.35)	0.397(0.31)	0.349(0.28)	0.046(0.09)	0.051(0.09	0.055(0.1)	0.040(0.090)	0.046(0.09)	0.050(0.09)
200-499	139 280	141 276	138 233	0.402(0.34)	0.328(0.29	0.277(0.24)	0.027(0.05)	0.028(0.05)	0.029(0.05)	0.021(0.047)	0.023(0.04)	0.024(0.04)
500-999	228 259	231 215	226 852	0.383(0.33	0.306(0.28)	0.254(0.23)	0.021(0.03)	0.022(0.03)	0.023(0.03	0.016(0.033)	0.017(0.03)	0.017(0.03)
1 000-1 999	447 448	453 482	442 903	0.374(0.33)	0.297(0.27)	0.245(0.22)	0.020(0.03)	0.021(0.03)	0.021(0.03)	0.014(0.029)	0.015(0.02)	0.015(0.02)
2 000-4 999	1 290 593	1 303 873	1 274 310	0.366(0.33)	0.289(0.27)	0.238(0.22)	0.018(0.03)	0.019(0.03)	0.020(0.03)	0.013(0.027)	0.014(0.02)	0.014(0.02)
5 000-9 999	2 010 370	2 027 558	1 982 159	0.358(0.33)	0.280(0.27)	0.229(0.21)	0.017(0.02)	0.018(0.02)	0.018(0.02)	0.011(0.025)	0.012(0.02)	0.013(0.02)
10 000-11 999	760 396	765 970	749 864	0.354(0.33)	0.275(0.26)	0.225(0.21)	0.016(0.02)	0.017(0.02)	0.017(0.02)	0.011(0.023)	0.012(0.02)	0.012(0.02)
12 000-14 999	1 100 143	1 106 024	1 081 484	0.352(0.33)	0.273(0.26)	0.223(0.21	0.016(0.02)	0.017(0.02)	0.017(0.02)	0.010(0.023)	0.011(0.02)	0.011(0.02)
15 000-16 999	708 390	711 382	695 392	0.349(0.33)	0.270(0.26)	0.221(0.21)	0.016(0.02)	0.017(0.02)	0.017(0.02)	0.010(0.022)	0.011(0.02)	0.011(0.02)
17 000-19 999	1 027 451	1 033 865	1 011 227	0.348(0.33)	0.269(0.26)	0.220(0.21)	0.016(0.02)	0.016(0.02	0.017(0.02)	0.010(0.022)	0.011(0.02)	0.011(0.02)
20 000-29 999	3 100 869	3 115 808	3 048 049	0.345(0.33)	0.265(0.26)	0.217(0.21)	0.015(0.02)	0.016(0.02)	0.016(0.02)	0.009(0.022)	0.010(0.02)	0.010(0.02)
30 000-49 999	4 710 189	4 739 668	4 629 739	0.341(0.33)	0.263(0.26)	0.214(0.2)	0.015(0.02)	0.015(0.02)	0.016(0.02)	0.009(0.021)	0.010(0.02)	0.010(0.02)
Non-syntenic SNPs	1 444 151	1 444 151	1 444 151	0.342(0.32)	0.258(0.25)	0.214(0.2)	0.0145(0.02)	0.015(0.02)	0.015(0.02)	0.008(0.020)	0.009(0.02)	0.009(0.02)

**Figure 2:**
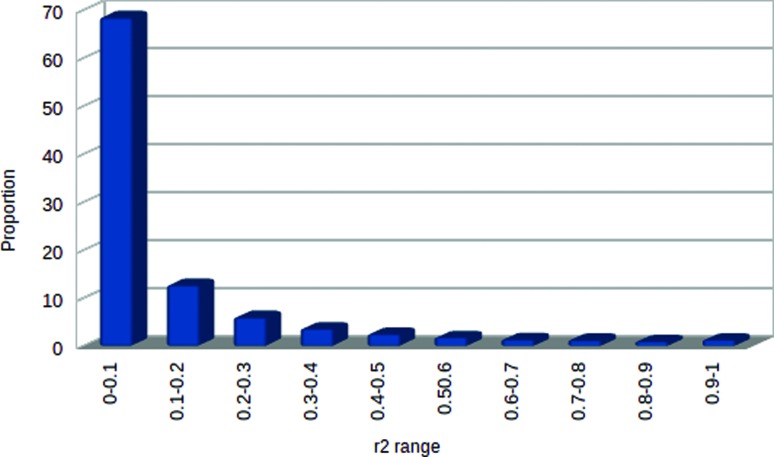
Distribution of *r*^2^ values between adjacent SNP pairs pooled over all autosomes.

The effect of MAF on the extent of LD among syntenic SNPs was studied using three different MAF thresholds: 1, 5, and 10% (Figure 1 and Table 1). Overall, we analyzed 17,056,201 SNP pairs for MAF=0.01, 17,164,817 for MAF=0.05, and 16,813,297 SNP pairs for MAF=0.1. It seemed that the MAF had a strong effect on mean *r*^2^ over short physical distances (≤ 200 Kb), while its effect on *D’* was more pronounced over long distances (Figure 1 and Table 1). For instance, within the range 0-19 Kb, mean *r*^2^ increased by 28% passing from MAF=0.01 to MAF=0.1, while the increase was only 8% within the 500-999 Kb interval. The range of *D’* and *r*^2^ values for SNP pairs more than 500 Kb apart were similar to those observed for non-syntenic markers, for which around 79% of *D’* values were lower than 0.4 and 94% of *r*^2^ values were lower than 0.05 (Figure S4). The distribution of *r*^2^ values between adjacent SNP pairs, combined over all autosomes, is shown in Figure 2. Most adjacent markers (~80%) had an average *r*^2^ value <0.2 while only 2% were in strong or complete LD (0.8 <*r*^*2*^ <1). Mean genomic distance between these marker pairs was 73.4 Kb and 35.4 Kb, respectively (data not shown). The mean value of *r*^2^ between adjacent SNP pairs pooled over all autosomes was 0.132 ± 0.19. Some variations in the LD values between adjacent SNPs were observed within chromosomes (Table 2), with BTA 19, 23, 25, 26, 28, and 29 having the lowest LD values and BTA 1, 6, and 8 having the highest ones. Per-chromosome values ranged from 0.106 ± 0.17 for BTA28 to 0.158 ± 0.228 for BTA06 (*r*^2^) and from 0.494 ± 0.33 for BTA23 to 0.158 ± 0.22 for BTA06 (*D’*). Unsurprisingly, chromosomes with the lowest LD values tended to have the lowest proportion of marker pairs with strong and moderate LD.

**Table 2:** Mean *r*^*2*^ and *D’* between adjacent SNPs on each chromosome in Tunisian native cattle and percentage of marker pairs with *r*^*2*^ >0.2 or 0.8.

BTA	Intermarker average distance (Kb)	Average D’ ± sd	Average r^2^ ± sd	% *r*^2^ >0.2	% *r*^2^ >0.8
1	71.47 ± 64.51	0.583 ± 0.35	0.153 ± 0.21	21.61	2.82
2	76.82 ± 78.74	0.573 ± 0.35	0.143 ± 0.20	19.81	2.64
3	74.60 ± 71.88	0.574 ± 0.35	0.141 ± 0.20	20.17	2.22
4	75.29 ± 64.98	0.572 ± 0.35	0.134 ± 0.20	18.57	2.27
5	86.16 ± 86.86	0.553 ± 0.35	0.127 ± 0.19	17.44	2.25
6	71.51 ± 79.12	0.583 ± 0.35	0.158 ± 0.22	21.93	3.40
7	77.68 ± 97.71	0.564 ± 0.35	0.144 ± 0.20	19.82	2.68
8	73.45 ± 63.08	0.579 ± 0.35	0.147 ± 0.21	20.47	2.65
9	81.52 ± 79.84	0.57 ± 0.35	0.141 ± 0.20	18.22	3.02
10	74.76 ± 110.10	0.555 ± 0.35	0.140 ± 0.20	19.74	2.89
11	75.18 ± 72.59	0.552 ± 0.35	0.143 ± 0.21	19.81	3.24
12	83.46 ± 135.71	0.551 ± 0.35	0.127 ± 0.19	18.27	1.74
13	72.71 ± 65.71	0.563 ± 0.35	0.131 ± 0.18	17.87	1.77
14	71.59 ± 66.72	0.563 ± 0.35	0.142 ± 0.20	19.50	2.44
15	77.33 ± 70.82	0.558 ± 0.34	0.130 ± 0.19	17.71	2.08
16	74.79 ± 78.75	0.565 ± 0.35	0.139 ± 0.20	20.40	2.05
17	73.13 ± 77.88	0.547 ± 0.35	0.128 ± 0.19	18.00	1.95
18	78.10 ± 81.84	0.549 ± 0.35	0.136 ± 0.20	19.66	2.26
19	69.24 ± 63.14	0.529 ± 0.35	0.116 ± 0.18	16.63	1.34
20	70.29 ± 60.83	0.559 ± 0.35	0.134 ± 0.20	18.19	2.29
21	77.96 ± 86.18	0.554 ± 0.35	0.139 ± 0.19	21.37	1.76
22	73.13 ± 64.58	0.549 ± 0.35	0.126 ± 0.19	18.06	1.76
23	73.63 ± 75.66	0.494 ± 0.34	0.108 ± 0.17	14.46	1.23
24	74.13 ± 65.68	0.541 ± 0.35	0.129 ± 0.19	16.70	2.22
25	68.39 ± 56.42	0.506 ± 0.34	0.122 ± 0.17	17.58	1.01
26	72.89 ± 61.70	0.521 ± 0.35	0.109 ± 0.17	13.47	1.38
27	71.65 ± 71.04	0.529 ± 0.35	0.122 ± 0.19	16.38	1.53
28	72.90 ± 62.37	0.51 ± 0.34	0.106 ± 0.17	13.72	1.37
29	76.08 ± 78.60	0.526 ± 0.35	0.110 ± 0.17	14.02	0.99
Mean	74.82 ± 75.62	0.551 ± 0.35	0.132 ± 0.19	18.26	2.11

### Effective population size

**Figure 3:**
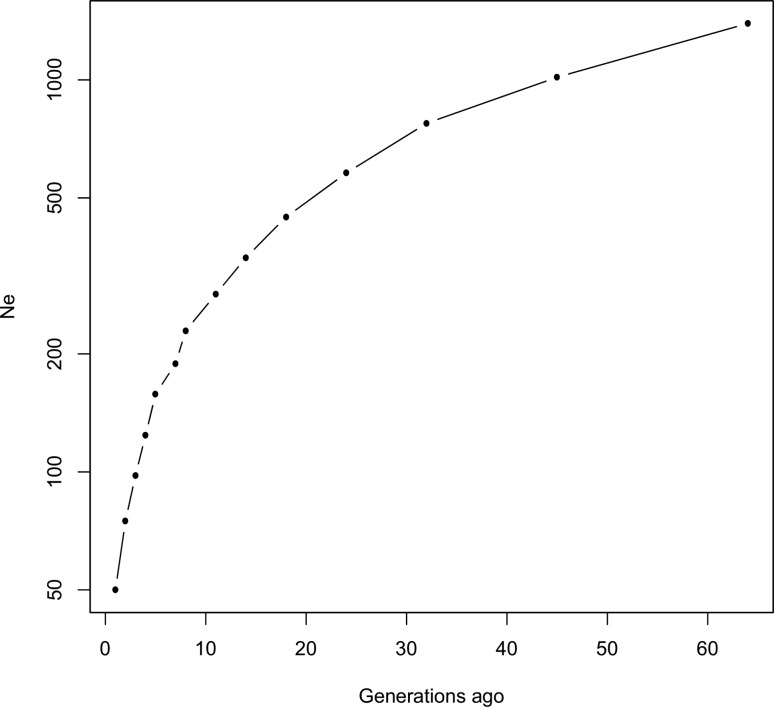
Average-estimated effective population size in the Tunisian cattle population, plotted against average generations in the past, truncated at 64 generations. Estimated effective population size was plotted on a log scale.

Average effective population size over the past 64 generations was estimated from the mean *r*^2^ values for the 29 bovine autosomes using the three MAF thresholds (Table 3). Figure 3 illustrates an example for MAF 0.1. Similar Ne estimates were found between the MAF 0.1 and the MAF 0.05 datasets. However, between these two datasets and the MAF 0.01 dataset, we observed discrepancies that increased over generations. In general, we observed a continual decrease in Ne across generations, with the average value falling from 1410 to 51 after 64 generations. It appeared that Ne declined slowly between 64 and 32 generations ago, and then this decline underwent a sudden acceleration from eight generations ago to the present, when Ne dropped from an average of 232 to an average of 191 (Table 3).

**Table 3:** Average estimated effective population size truncated at 64 generations for three different MAF thresholds.

Generations ago	Ne_MAF0.1	Ne_MAF0.05	Ne_MAF0.01	Mean Ne
1	50	51	52	51
2	75	75	78	76
3	98	98	101	99
4	124	124	127	125
5	158	159	163	160
7	189	190	195	191
8	229	230	237	232
11	284	284	292	287
14	352	354	366	357
18	447	450	463	453
24	579	577	597	584
32	775	770	799	781
45	1016	1023	1053	1031
64	1393	1397	1440	1410

## Discussion

In the present study, we first identified runs of homozygosity in a sample of 87 Tunisian cattle individuals. Characterization of ROHs, particularly their size and frequency, provides information about relatedness within a population. Our findings showed that more than one sixth of the individuals in our sample had a total sum of ROHs >250 Mb, with almost all individuals having at least one ROH >25 Mb. Because long ROH fragments are indicators of recent inbreeding, we can hypothesize that consanguineous mating is quite common in the local livestock farming system. This is particularly problematic as long autozygous ROHs are generally associated with an increased incidence of strongly deleterious mutations as well as mildly deleterious variants (Szpiech *et al.*, 2013). However, the mean number of ROHs per individual observed for native Tunisian cattle (38 ± 12.6) was low compared to that found in other European cattle breeds. For instance, Ferencakovic *et al.* (2013) reported that Brown Swiss, Tyrol Grey, and Pinzgauer cattle had, on average, 94.76 ± 14.55, 70.86 ± 9.51, and 59.96 ± 9.91 ROHs per individual, respectively. This most likely reflects differences in the history of breed formation between European breeds and Tunisian cattle, with the former originating from a more recent bottleneck and subjected to a more pronounced selection pressure.

Next, we aimed to assess the extent of genome-wide LD and to infer the effective population size in the native Tunisian cattle population using medium-density SNP chips and two of the most popular metrics for quantifying LD: *D’* and *r*^2^. Our results revealed a gradual decline in these two parameters over increasing inter-marker distances, with a steeper rate of decay for *r*^2^ than for *D’*. Furthermore, we observed an inflation in estimates of *D’* (which was more noticeable over long inter-marker distances) with a decrease in the MAF threshold used. This finding has previously been reported in other studies (e.g., Khatkar *et al.*, 2008; Bohmanova *et al.*, 2010; Espigolan *et al.*, 2013) and might be explained by the fact that, in the formula for *D’*, the denominator is set to the minimal product of SNP allele frequencies and will be lower when the difference between allele frequencies is higher, thus leading to over-estimation of *D’*. Khatkar *et al.* (2008) reported that the observed inflation in *D’* estimates with an increase in the proportion of rare alleles is mainly due to the fact that rare alleles are, in general, younger than common alleles, and hence may still be in LD. Instead, mean values of *r*^2^ dropped noticeably as the MAF threshold was lowered at all inter-marker distances.

Correction for sample size resulted in only a small change in *r*^2^ estimates at short inter-marker distances, indicating that 87 individuals were sufficient to estimate *r*^2^ with acceptable reliability. However, this number may be insufficient for a correct estimate of *D’*, because small samples may fail to sample rare fourth gametes which, therefore, can inflate *D’* (Khatkar *et al.*, 2008). Indeed, Bohmanova *et al.* (2010) reported that a minimal sample size of 55 animals was required for accurate estimation of *r*^2^, while 444 animals were required for *D’*, and other studies have likewise reported that *D’* is more sensitive to sample size variations than *r*^*2*^ (e.g., Ardlie *et al.*, 2002; Du *et al.*, 2007). We therefore considered our estimates of *r*^2^ to be more reliable than the *D’* estimates.

In order to further reduce the effect of sampling bias on *r*^*2*^ estimates in our study, it would be reasonable to consider only values obtained using a MAF threshold of 0.1. This is because LD around common alleles (those with a moderate-to-high frequency) can be measured with a modest sample size (80 ± 100 chromosomes) to a precision within 10 ± 20% of the asymptotic limit (Reich *et al.*, 2001). Around 74% of the SNPs in the MAF 0.1 dataset had a moderate-to-high MAF (>0.2) (Figure S1), making the *r*^2^ estimates that were based on this dataset robust over short inter-marker distances.

Differences in LD were observed between chromosomes and might result from variations in chromosome length, with longer chromosomes having higher LD than shorter ones. Indeed, we found a high correlation (~78%) between *r*^2^ value and chromosome length. In agreement with our results, Stapley *et al.* (2010) found that LD extended further on the macrochromosomes than on microchromosomes in the zebra finch genome. Another possible explanation for inter-chromosomal variations in LD might be the difference in total sum of ROHs observed between high- and low-LD chromosomes (Table S3). This hypothesis is corroborated by the high positive correlation (~82%) found between LD and total sum of ROHs per chromosome. Similarly, studies of LD in humans have reported that ROHs are more common in regions with high linkage disequilibrium and a low recombination rate (e.g., Gibson *et al.*, 2006).

**Figure 4:**
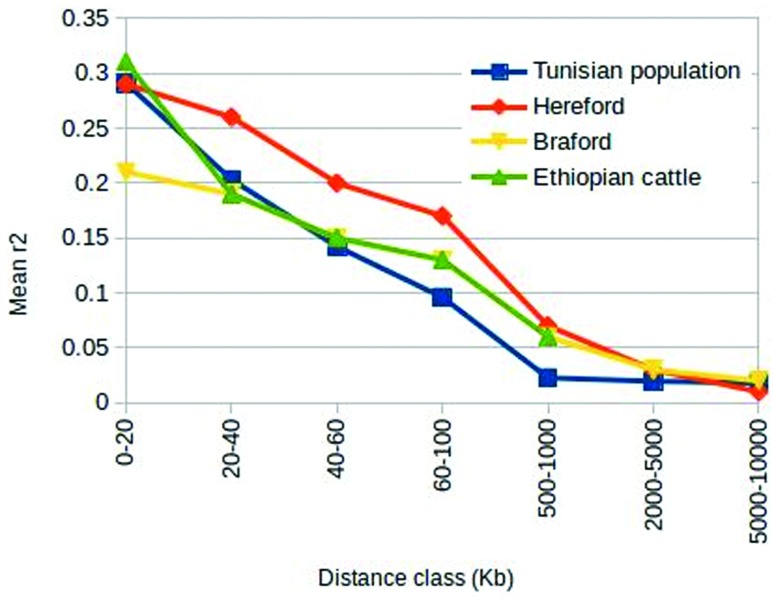
Decay in linkage disequilibrium (*r*^2^) over increasing inter-marker distance in Tunisian, Hereford, Braford, and local Ethiopian cattle.

Next, we compared our results with those obtained from other taurine breeds using a similar SNP panel. We found that LD decay in the Tunisian population was faster than that reported by Biegelmeyer *et al.* (2016) for a sample of 391 Braford and 2079 Hereford cattle, as well as that published by Edea *et al.* (2014) for Ethiopian local cattle (Figure 4). Qanbari *et al.* (2010) found that, in German Holstein cattle, mean *r*^2^ decreased from 0.30 between marker pairs separated by less than 25 Kb to 0.12 for those separated by 100-200 Kb; this decrease was slower than that observed in our sample (0.29 to 0.05 for Tunisian native cattle over the same change in physical distance). Instead, in reporting LD estimates for a sample of 887 North American Holstein bulls, Bohmanova *et al.* (2010) found that mean *r*^2^ for markers 40-60 kb apart was 0.20, which is higher than that found in our study (0.14) for the same inter-marker distance. However, it is difficult to compare our results directly with these previous studies due to differences in the scale of sampling, given that the level of LD decreases with increasing sample size (Hill and Robertson, 1968). When we restricted our comparisons to studies with smaller sample sizes, we found that the mean *r*^*2*^ value for adjacent markers in the present study (pooled over all autosomes; mean = 0.132, range: 0.106 to 0.158) was lower than those reported by Flury *et al.* (2010) for 128 indigenous Swiss cattle (mean *r*^*2*^ = 0.24, range: 0.19 to 0.26), by Mastrangelo *et al.* (2014) for 76 Italian Modicana individuals (mean *r*^*2*^ = 0.200, range: 0.168 to 0.234), and by Beghain *et al.* (2013) for 30 Blonde d’Aquitaine bulls (mean *r*^*2*^ = 0.205, range: 0.172 to 0.241). In summary, although our results are based on a smaller sample size, they paint a coherent picture: the lower level of genome-wide LD and its faster decay in Tunisian cattle compared to other breeds is consistent with the absence of strong selective pressure and with the exclusive use of natural reproductive methods within this population (unlike Holstein, Hereford, or Braford breeds). This hypothesis is further corroborated by the fact that mean *r*^2^ values between adjacent SNPs for the artificially unselected Italian breed Cinisara were similar to those reported in our study (mean *r*^*2*^ = 0.168, range: 0.138 to 0.184; Mastrangelo *et al.*, 2014)*.*

We used average *r*^2^ estimates (combined over all autosomes) at different inter-marker distances to infer past effective population sizes of the local Tunisian cattle population. Short inter-marker distances reflect the effective population size over historical periods of time while long inter-marker distances give information on population size in the immediate past (Hill, 1981). Using a MAF threshold of 0.01 tends to increasingly overestimate Ne across generations, while a MAF threshold of 0.05 is less subject to erroneous estimates of effective population size. Our study revealed that the Tunisian cattle population has undergone a sudden, substantial drop in Ne, starting from eight generations ago. If we assume a generation time for local cattle of 5 years, this period corresponds to the mid-1970s, and indeed, it was reported that the numbers of local and cross-bred cattle in Tunisia fell by 32% between 1975 and 1990 because of the massive introduction of European breeds (Kayouli, 2001). In addition, it is worth noting that, due to the small sample size in our study, it is likely that the values we found for recent Ne underestimate the true Ne in the whole native Tunisian cattle population; according to Corbin *et al.* (2012), when Ne is assumed to be variable, a severe underestimation occurs in recent Ne estimates. Therefore, the inferences for recent Ne in the present study should be considered cautiously due to the sampling bias. Taken together, the pattern of ROHs and the low levels of long-range LD found in the present study, combined with the relatively high levels of genetic diversity reported in a previous study (Ben Jemaa *et al.*, 2015) may indicate that native Tunisian cattle have not been subjected to strong genetic drift. We thus hypothesize that there has been a significant drop in the effective population size of Tunisian cattle over successive generations, but with higher real Ne values than those found in our study (especially regarding recent Ne estimates).

The fact that the recent effective population size that was inferred here for local Tunisian cattle was small implies that this population is endangered. According to Franklin (1980), populations with Ne <50 are considered to be at immediate risk of extinction. Other authors have gone even further, suggesting that a threshold of Ne = 50 is too small to ensure long-term population survival. For instance, Meuwissen (2009) reported that a threshold of Ne = 100 would be necessary to ensure the long-term viability of an animal population. This is because such small populations are exposed to loss of genetic variation through high levels of both inbreeding and genetic drift, which can substantially increase the extinction probability of populations in changing environments.

In conclusion, this work presents the first assessment of ROH patterns and LD distribution at the genome-wide level in the native Tunisian cattle population. Compared to European breeds, we found that Tunisian cattle had less LD, and that it decayed faster with physical distance between markers. Therefore, a higher SNP density might be necessary to carry out effective genome-wide association mapping within this population. We provide evidence of a rapid decline in the effective population size in Tunisian cattle, associated with high levels of recent inbreeding within a significant proportion of individuals. These two observations indicate an urgent need to establish a conservation plan that includes a well-designed genetic management program for this population.

## Supplementary material

The following online material is available for this article:

Figure S1 Distribution of allele frequencies for the MAF 0.1, MAF 0.05, and MAF 0.01 datasets (syntenic SNPs).

Figure S2 The number of runs of homozygosity (ROHs) per chromosome (bars) in the 87 Tunisian cattle individuals.

Figure S3 Total sum of ROHs in the individual genomes of the 87 Tunisian cattle individuals.

Figure S4 Frequency distribution of LD estimates between non-syntenic pairs of SNPs.

Table S1 Description of SNP distribution per chromosome for three different MAF thresholds.

Table S2 Repartition of ROH categories from the 15 individuals that had a total sum of ROH >250 Mb.

Table S3 Total sum of ROHs per chromosome on high-LD vs low-LD chromosomes.
