# Supplementary material for: Linkage disequilibrium and past effective population size in native Tunisian cattle
Source: Genet Mol Biol. 2019 Feb 18;42(1):52–61. doi: 10.1590/1678-4685-GMB-2017-0342 (PMC6428135; doi:10.1590/1678-4685-GMB-2017-0342)
Supplement: Supplementary file 1 [file 1415-4757-GMB-1678-4685-GMB-2017-0342-20190130-suppl5.pdf]

Supplementary Material to "Linkage disequilibrium and past effective population size in native Tunisian cattle"

Table S1 - Description of SNP distribution per chromosome for three different MAF thresholds in the Tunisian population.

BTA	SNP number			Marker coverage(Mb)			SNPs/Mb			Intermarker average (sd) (Kb)		
	**MAF 0.1**	**MAF 0.05**	**MAF 0.01**	**MAF 0.1**	**MAF 0.05**	**MAF 0.01**	**MAF 0.1**	**MAF 0.05**	**MAF 0.01**	**MAF 0.1**	**MAF 0.05**	**MAF 0.01**
1	2213	2230	2196	158.09	158.09	158.03	14	14	14	71.47±64.81	70.96±65.88	71.99±62.84
2	1775	1784	1781	136.66	136.66	136.66	13	13	13	77.04±80.8	76.65±79.12	76.78±76.31
3	1609	1639	1627	121.14	121.14	121.12	13	14	13	75.34±71.33	73.96±73.26	74.49±71.04
4	1561	1615	1627	120.42	120.45	120.39	13	13	14	77.2±68.14	74.63±62.7	74.04±64.1
5	1406	1409	1402	121.01	121.01	121.04	12	12	12	86.13±88.79	85.95±86.54	86.4±85.26
6	1652	1683	1661	119.01	119.01	119.01	14	14	14	72.09±81.26	70.76±77.34	71.7±78.77
7	1443	1446	1449	112.36	112.12	112.27	13	13	13	77.92±108.56	77.59±95.27	77.54±89.29
8	1524	1543	1548	112.91	112.91	112.91	13	14	14	74.14±64.79	73.22±63.6	72.99±60.86
9	1274	1302	1307	105.46	105.46	105.34	12	12	12	82.85±83.78	81.06±76.75	80.66±78.97
10	1374	1391	1405	103.18	104.17	104.17	13	13	13	75.15±109.22	74.95±111.95	74.2±109.13
11	1407	1426	1445	107.14	107.14	107.1	13	13	13	76.2±74.6	75.18±73.84	74.17±69.33
12	1060	1102	1108	90.85	90.85	90.85	12	12	12	85.79±145.79	82.52±140.73	82.07±120.6
13	1137	1169	1154	83.86	83.75	83.72	14	14	14	73.82±68.55	71.7±63.17	72.61±65.4
14	1171	1168	1148	83.15	83.15	83.1	14	14	14	71.07±66.46	71.25±68.07	72.45±65.63
15	1079	1092	1097	84.22	84.22	84.01	13	13	13	78.13±72.09	77.2±70.52	76.66±69.83
16	1090	1089	1083	81.25	81.25	81.25	13	13	13	74.61±80.48	74.68±81.39	75.09±74.4
17	1023	1030	1022	74.89	74.89	74.89	14	14	14	73.28±79.75	72.78±81.09	73.35±72.78
18	845	846	826	65.4	65.4	65.51	13	13	13	77.49±81.48	77.4±81.27	79.41±82.75
19	920	923	913	63.54	63.54	63.54	14	15	14	69.14±63.42	68.92±63.1	69.67±62.9
20	1022	1032	1005	71.59	71.59	71.59	14	14	14	70.12±62.23	69.44±59.01	71.31±61.24
21	917	913	899	71.1	70.31	71.1	13	13	13	77.62±97.15	77.09±79.87	79.17±81.51
22	836	832	842	61.1	61.12	61.12	14	14	14	73.17±67.86	73.55±63.57	72.68±62.31
23	705	719	699	52.1	52.1	51.88	14	14	13	74±77.34	72.56±74.33	74.33±75.31
24	836	847	834	62.05	62.14	62.14	13	14	13	74.32±64.09	73.46±66.48	74.6±66.46
25	627	630	621	42.8	42.8	42.62	15	15	15	68.38±57.6	68.05±56.27	68.75±55.39
26	697	703	705	50.91	50.76	51.54	14	14	14	73.15±61.83	72.31±60.5	73.21±62.77
27	638	632	630	45.33	45.33	45.25	14	14	14	71.17±71.3	71.84±70.49	71.94±71.34
28	636	642	625	46.18	46.15	46.15	14	14	14	72.73±61.62	72±61.75	73.96±63.74
29	669	672	674	51.1	51.1	50.87	13	13	13	76.5±84.71	76.16±76.45	75.59±74.64
Sum Average	33 146	33 509	33 333	86.17	86.16	86.18	13	13	13	75.17±77.92	74.41±75.32	74.89±73.62
