# Supplementary material for: Linkage disequilibrium and past effective population size in native Tunisian cattle
Source: Genet Mol Biol. 2019 Feb 18;42(1):52–61. doi: 10.1590/1678-4685-GMB-2017-0342 (PMC6428135; doi:10.1590/1678-4685-GMB-2017-0342)
Supplement: Supplementary file 2 [file 1415-4757-GMB-1678-4685-GMB-2017-0342-20190130-suppl1.pdf]

Supplementary Material to "Linkage disequilibrium and past effective population size in native Tunisian cattle"

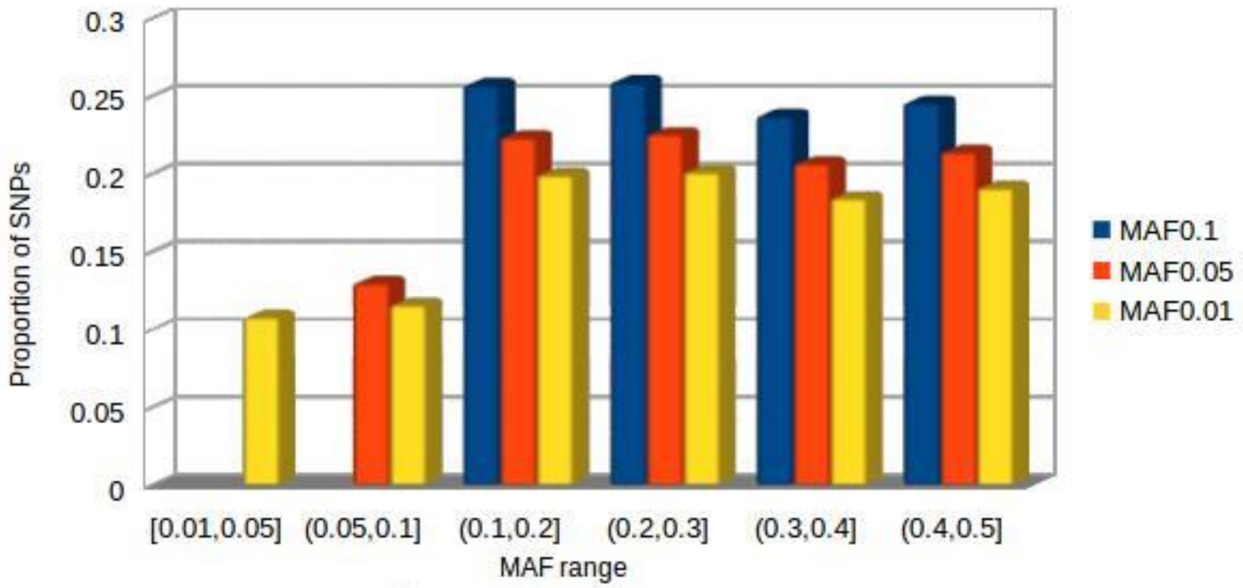

Figure S1 - Distribution of allele frequencies for the MAF 0.1, MAF 0.05, and MAF 0.01 datasets (syntenic SNPs).
