# Supplementary material for: Linkage disequilibrium and past effective population size in native Tunisian cattle
Source: Genet Mol Biol. 2019 Feb 18;42(1):52–61. doi: 10.1590/1678-4685-GMB-2017-0342 (PMC6428135; doi:10.1590/1678-4685-GMB-2017-0342)
Supplement: Supplementary file 3 [file 1415-4757-GMB-1678-4685-GMB-2017-0342-20190130-suppl2.pdf]

Supplementary Material to "Linkage disequilibrium and past effective population size in native Tunisian cattle"

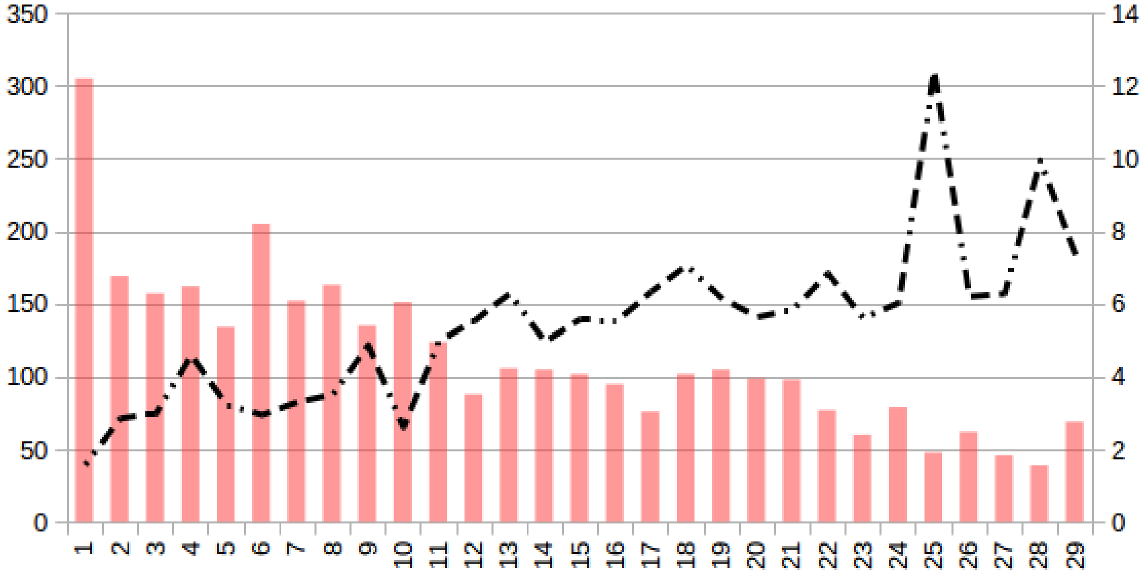

Figure S2 - The number of runs of homozygosity (ROHs) per chromosome (bars) in the 87 Tunisian cattle individuals.
