# Supplementary material for: Linkage disequilibrium and past effective population size in native Tunisian cattle
Source: Genet Mol Biol. 2019 Feb 18;42(1):52–61. doi: 10.1590/1678-4685-GMB-2017-0342 (PMC6428135; doi:10.1590/1678-4685-GMB-2017-0342)
Supplement: Supplementary file 5 [file 1415-4757-GMB-1678-4685-GMB-2017-0342-20190130-suppl6.pdf]

Supplementary Material to "Linkage disequilibrium and past effective population size in native Tunisian cattle"

Table S2 - Repartition of ROH categories from the 15 individuals that had a total sum of ROH > 250 Mb.

**Individual/ROH category (Mb)**	**[1,5]**	**[5,10]**	**[10,15]**	**[15,20]**	**[20,25]**	**[25,50]**	**[50,100]**
TUNPOP_001	35	7	3	0	1	1	1
TUNPOP_002	38	6	5	0	0	3	3
TUNPOP_003	47	9	3	2	1	0	0
TUNPOP_004	38	6	6	3	4	6	0
TUNPOP_005	55	7	9	3	2	5	0
TUNPOP_006	45	12	5	2	2	7	0
TUNPOP_007	31	5	2	2	1	7	3
TUNPOP_008	31	3	7	1	2	7	4
TUNPOP_009	17	6	4	3	1	8	2
TUNPOP_010	15	4	2	3	1	3	0
TUNPOP_011	25	10	5	5	2	6	0
TUNPOP_012	42	3	2	4	1	1	0
TUNPOP_013	32	4	6	2	1	5	1
TUNPOP_014	37	8	5	4	1	3	0
TUNPOP_015	49	5	3	0	2	2	1
