# Supplementary material for: Linkage disequilibrium and past effective population size in native Tunisian cattle
Source: Genet Mol Biol. 2019 Feb 18;42(1):52–61. doi: 10.1590/1678-4685-GMB-2017-0342 (PMC6428135; doi:10.1590/1678-4685-GMB-2017-0342)
Supplement: Supplementary file 7 [file 1415-4757-GMB-1678-4685-GMB-2017-0342-20190130-suppl7.pdf]

Supplementary Material to "Linkage disequilibrium and past effective population size in native Tunisian cattle"

Table S3 - Total sum of runs of homozygosity (ROH) per chromosome on high LD (BTA01, BTA06, BTA08) vs. low LD chromosomes (BTA19, BTA23, BTA25, BTA26, BTA28).

Chromosome	Total sum of ROH for all individuals (Mb)	r^2 value
BTA01	797.35	0.153 ± 0.21
BTA06	734.60	0.158 ± 0.22
BTA08	632.89	0.147 ± 0.21
BTA19	380.95	0.116 ± 0.18
BTA23	192.72	0.108 ± 0.17
BTA25	223.72	0.122 ± 0.17
BTA26	209.53	0.109 ± 0.17
BTA28	175.90	0.106 ± 0.17
